# Measles Outbreak in Socioeconomically Diverse Sections: A Review

**DOI:** 10.7759/cureus.62879

**Published:** 2024-06-21

**Authors:** Ruchira Lanke, Vilas Chimurkar

**Affiliations:** 1 Medicine, Jawaharlal Nehru Medical College, Datta Meghe Institute of Higher Education and Research, Wardha, IND; 2 Anatomy, Jawaharlal Nehru Medical College, Datta Meghe Institute of Higher Education and Research, Wardha, IND

**Keywords:** measles epidemiology, measles prevention, measles treatment, measles diagnosis, measles transmission, measles outbreak, measles

## Abstract

Measles outbreaks among socioeconomically weaker sections constitute a significant public health challenge. The objective is to highlight the specific vulnerabilities and contributing factors that make these communities more susceptible to measles outbreaks. Socioeconomically weaker sections, often characterized by poverty, inadequate healthcare access, overcrowding, and suboptimal immunization rates, face heightened risks of measles outbreaks. These outbreaks occur due to various interrelated factors, including limited healthcare infrastructure, low vaccine coverage, a lack of awareness about vaccination benefits, and difficulties in accessing healthcare services. The effects of measles outbreaks in socioeconomically disadvantaged areas are critical. Particularly among vulnerable groups, including newborns, expectant mothers, and malnourished people, measles increases morbidity and mortality. There is also a considerable financial impact on the healthcare system and the afflicted families. Measles outbreaks in these populations must be addressed using a diversified strategy. In order to improve vaccine coverage through targeted immunization programs, raise community vaccination awareness, and address social determinants of health, efforts should concentrate on bolstering the healthcare infrastructure. Effective epidemic control and prevention depend on coordinated efforts by healthcare practitioners, legislators, local leaders, and public health groups. Healthcare systems can lessen the impact of measles in socioeconomically disadvantaged areas and strive toward attaining equitable health outcomes for all populations by addressing the socioeconomic variables that lead to the vulnerability of measles.

## Introduction and background

More than 100,000 people die from measles yearly, down from more than 2 million before the introduction and widespread use of the measles vaccine. Measles is a highly contagious disease caused by infection with the measles virus. The respiratory technique for transmitting the measles virus causes fever, coughing, coryza, and conjunctivitis, followed by the recognizable rash. Most organ systems are affected by measles complications, with pneumonia becoming the most frequently reported cause of morbidity and death linked to measles [1].

Although mortality has been declining over the past 50 years, measles is still a significant reason for child mortality, which can be avoided through vaccination. The World Health Organization (WHO) launched the Expanded Programme on Immunization (EPI) in 1974 to boost childhood immunization rates against a variety of diseases that can be prevented by vaccines, such as measles [2]. In Vietnam, routine measles vaccination for infants below one year of age gained popularity in 1985, following the establishment of the EPI in 1981 in an effort to combat child mortality in a socioeconomically underdeveloped country such as Vietnam [3]. The laboratory diagnosis mainly depends on the identification of particular antibodies called IgM in serum, dried-out blood pigmentation, or oral water, or the identification of viral ribose nucleic acid (RNA) in urine, oral fluid, throat or nasal cavity swabs, or dried blood spots [4]. In India, measles outbreaks were observed in hilly areas under the supervision of two subcenters, Saili and Sarah, despite the implementation of high immunization programs [5]. The causes of this pandemic are many. In this case, ignorance and knowledge are complementary. Many important criteria, such as a remote location, low socioeconomic level, poverty, and illiterate mothers, increased the likelihood that people would believe in conventional, illogical causes and effects. In the past, people believed that measles was a goddess's curse; thus, despite having a high vaccination rate, they preferred traditional faith treatments [6].

In Bihar's Madhepura district, in the village of Puraini, there is yet another instance of a measles outbreak. A prevalence study was carried out among children between six months and 12 years. Measles cases prompted house-to-house visits and inspections. The main finding of the survey was that the lack of immunization contributed to the measles outbreak among the youngsters in the Musahar neighborhood. It is important to take the strengthening of the healthcare delivery system seriously [7]. With approximately 164,000 fatalities attributable to measles in 2008, vaccination has significantly contributed to the remarkable success of lowering the number of measles deaths [8]. According to studies carried out in the United States, measles vaccination refusal and underuse might be grouped and associated with other social factors impacting health inequalities [9]. Despite reliable and effective vaccination, measles continues to be a significant cause of pediatric morbidity and death worldwide [10]. The percentage of one- to two-year-old children who had received a measles vaccination was calculated for each survey period and was broken down by sex, location, ethnicity, mother's educational level, and socioeconomic situation. Measles vaccination rates between 2014 and 2000 were compared, and a 95% confidence interval was shown. Between 2000 and 2011, the percentage of measles vaccinations increased dramatically, but between 2011 and 2014, it slightly decreased. The measles vaccination rate in 2014 was 85.6%.

In contrast to their socially and economically aided counterparts in Vietnam, we discovered that women from ethnic groups who were poorer and less educated before 2014 were less likely to have taken advantage of the chance to have their children immunized. Vietnam's susceptibility to measles outbreaks increased as rates dropped in 2014 to 85.6% and 82.4% in urban areas [11]. Measles is viewed as a contender for eradication due to the accessibility of a very efficient and reasonably priced vaccine, the monotypic form of the virus, and the absence of an animal reservoir [12]. The present review article aims to identify the prevalence of measles in socioeconomically diverse sections throughout the world.

## Review

Methodology

**Figure 1:**
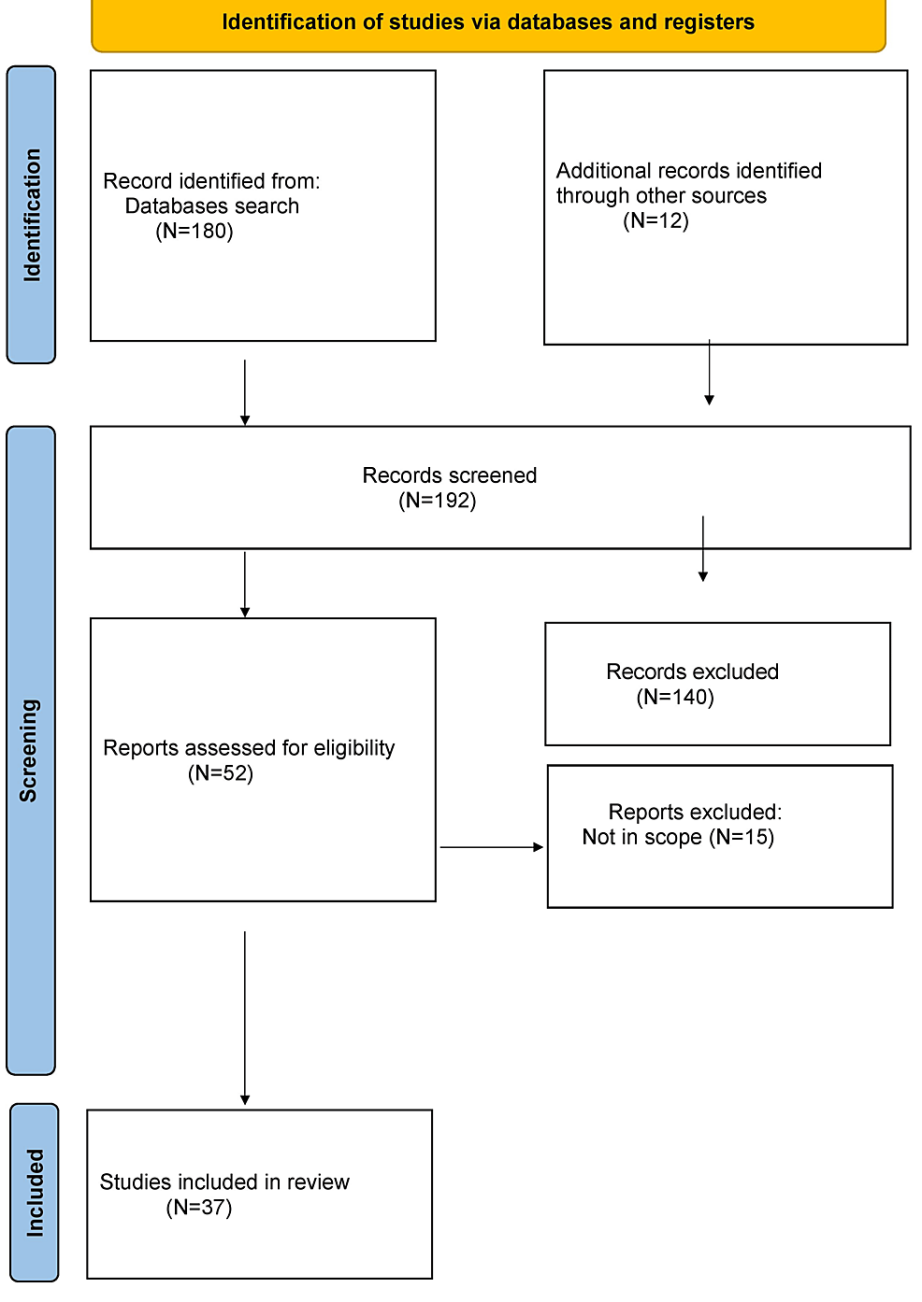
Selection process of the articles included in this study Image credit: Ruchira Lanke

An extensive literature review was conducted using databases such as PubMed. Keywords such as "measles," "measles outbreak," "children," "prevalence," "epidemic," "vaccination," "immunization," and "symptoms" were used to refine the search. Research that examined the reasons for the increasing prevalence of measles in children was included. The inclusion of the studies is depicted in Figure 1.

Measles outbreak in underdeveloped countries

Outbreak in Niger

In Niger, a total of 50,138 cases of measles were reported in 2003 (21 years ago from 2024), with 201 deaths (case fatality rate (CFR), 0.4%). The WHO aimed to investigate the hygiene and actual CFR of the measles in the Mirriah district of Niger. The investigation participants were from 22 communities in the Mirriah district that had reported occurrences of measles. A case of measles was defined as a sickness with a fever, rash, cough, coryza, or conjunctivitis, with the rash appearing between January 1 and April 15, 2003. Unless there was clear evidence of another cause, deaths that occurred within 30 days of the commencement of the rash were generally attributed to measles. According to our analysis, the measles CFR in the Mirriah district may be 20-fold greater than the estimate determined from routine surveillance and two times higher than the WHO regional estimate. Wide-ranging vaccination efforts, improved routine vaccination services, and recurring investigation programs will all be necessary to lower measles mortality in Niger [13].

Measles outbreak in developing countries

The measles virus belongs to the *Paramyxoviridae* family of viruses and is responsible for causing the highly contagious disease known as rubeola, which is also vaccine-preventable. The pathognomonic enanthem (Koplik's spots), fever, runny nose, cough, classic erythematous, maculopapular rash, diarrhea, headache, sensitivity to light, sore throat, skin rash are usually the first symptoms of the sickness. The WHO advises that in countries with high rates of measles transmission, it is recommended to administer two doses of the measles vaccine between nine months and 15-18 months, respectively [14]. The signs and symptoms of measles are presented in Table 1.

**Table 1:** Signs and symptoms of measles Source: [14]

Signs and Symptoms		
Fever	Dry cough	Headache
Runny nose	Skin rash	Koplik’s spots
Cough	Pink rash	Sensitivity to light
Loss of appetite	Diarrhea	Sore throat

*Outbreak in India*

In addition to its ongoing measles-rubella Supplementary Immunization Activity (SIA) program, Nepal's National Immunization Program (NIP) launched an outbreak response immunization (ORI) campaign in 2020. Both campaigns were put into action while the COVID-19 transmission was still ongoing. By April, eight districts in Nepal had reported 220 confirmed cases of measles, along with two fatalities. In order to make "Go/No-Go" judgments for ORI interventions, the NIP survey data includes observations (measles and COVID-19), measles booster performance and immunity profile, program capacity, and group engagement. It then applied a logical decision-making framework to the compiled data. The National Immunization Advisory Committee (NIAC) granted its seal of approval following a thorough evaluation. This was an illustration of a workable use of the global framework for carrying out a widespread immunization campaign during COVID-19 using a straightforward, logical decision-making framework [15].

The study was conducted in the Puraini village in December 2008 during a measles outbreak among children aged six months to 12 years. Active measles cases were defined according to WHO case definition standards. Pre-structured questionnaires were used to gather information on the cases' demographics, immunization history, and disease outcomes. Blood samples from five cases were submitted for laboratory confirmation. A total of 52 cases, eight fatalities, a 28% attack rate, and a 15.4% CFR were reported. Dysentery with pneumonia was the most prevalent post-measles consequence, accounting for 35% of patients. All five of the serum samples provided for confirmation of the anti-measles IgM antibody results were positive. The lack of knowledge, doubt about vaccination, and lack of access to medical personnel were the reasons why no child had ever received the measles vaccine in the past [16].

The health department in Konsa village, a remote village on the Indo-Myanmar border, reported four probable measles-related deaths on May 23, 2017, in Longding district, Arunachal Pradesh, India. Measles was classified as a case in which a resident of the hamlet of Longding district experienced fever, a maculopapular rash, cough, coryza, or conjunctivitis between March 1 and June 18, 2017. Due to insufficient access to healthcare, this outbreak was probably caused by low MCV1 and vitamin A coverage. Following the investigation, a district-wide measles catch-up effort and routine immunization were resumed [17].

Outbreak in Kenya

Despite the availability of a highly effective measles vaccine and a global reduction in measles mortality by over 79% since 2000, measles remains a significant cause of vaccine-preventable deaths worldwide. Africa has played a crucial role in the global effort to control measles. However, Kenya has faced numerous challenges in combating measles since the beginning of the 21st century. Nevertheless, they have received support through innovative developments. Efficiently adjusting plans and managing additional measles immunization initiatives according to up-to-date evidence could be crucial for boosting coverage in future campaigns. Recognizing the need to address rubella alongside measles, Kenya introduced the measles and rubella (MR) vaccine in a statewide SIA campaign in 2016. In 2009 and 2012, during the SIAs conducted, Red Cross volunteers conducted door-to-door visits to raise awareness about vaccination and collect community data on vaccination coverage. In managing outbreaks, various techniques such as case-based surveillance, detection of measles-specific IgM antibodies, and real-time PCR were used to track and confirm measles infections. Kenya also explored alternative survey methods using dried blood spots and urine samples. Seroepidemiological surveys of measles surveillance are primarily utilized in developed countries, whereas in developing countries, these surveys are employed to identify specific risk groups with low immunity levels [18].

Measles in developed countries

Outbreak in Europe

In Europe, particularly in France, there has been a measles outbreak since 2008. If healthcare workers (HCWs) are not safeguarded, they could become ill and spread disease to patients. A total of 351 HCWs took part in a survey-based investigation that was carried out in three medical centers at universities in Paris between April and June 2011. At the time of enrollment, information regarding age, the healthcare system, employment, prior measles infection and immunization, prior measles serology tests, and readiness to get a measles vaccination in the event of seronegativity were all gathered. A total of 8.3% of the HCWs in this cohort were susceptible to measles, with individuals under 30 making up the majority of the age group. The measles vaccine was well-tolerated. A vaccination campaign in a medical context should concentrate on junior HCWs and students [19].

The European Region (EUR) countries of the WHO reported a record-high number of measles infections (37 deaths) from January 2018 to June 2018. The leading cause of the rise in incidence is inadequate immunization rates in prior years. The majority of cases were recorded from Ukraine, although there were also significant numbers reported from France, Georgia, Greece, Italy, the Russian Federation, and Serbia. Europe is the most popular travel destination in the world and is typically regarded as having low risks of infectious diseases. Because of this, travelers might not factor the importance of a pretravel medical checkup, including immunization, into their pre-departure planning. Measles is highly transmissible, and the WHO EUR's high number of cases increases the probability for US residents who are not traveling but come into proximity with sick returning travelers, in addition to putting at-risk unvaccinated and inadequately vaccinated visitors. Travelers are advised by the United States Centers for Prevention and Control of Diseases to be aware of the measles virus's spread in Europe and to acquire all recommended vaccinations, particularly the measles vaccine. Healthcare practitioners should have an elevated degree of concern for measles when patients with close contact with foreign tourists or travelers leaving Europe present with a febrile rash illness. It is important to remember this because of the continuing outbreak of the disease in the WHO EUR [20].

The WHO has set a goal to eliminate measles and rubella by 2015 in the WHO EUR. To confirm the end of transmission, it is necessary to demonstrate the absence of endemic measles virus (MV) through genotyping data. To assess the level of MV circulation in a specific area of the EUR, we examined the transmission chains of the most significant MV variants reported in Central and Continental Western Europe (CCWE) from 2006 to 2013. Several countries, including Switzerland, France, Bulgaria, and Romania, experienced large measles outbreaks, resulting in thousands of cases. The prolonged presence of a particular MV variant for more than a year during these outbreaks indicated ongoing transmission within the population. The circulation periods of the four most prevalent MV types in the entire CCWE region ranged from 18-44 months. Achieving the goal of eradicating measles is challenging due to persistent MV transmission, particularly affecting unvaccinated individuals in hard-to-reach populations and the general public. Further efforts are required to attain the EUR's eradication objective [21].

*Outbreak* *in Japan*

One of the crucial requirements to be confirmed in order to eliminate measles is genotyping evidence that indicates the cessation of endemic MV transmission. Since 2014, the majority of the measles cases at prefectural public health centers across Japan have undergone MV genotype analyses. In March 2015, Japan's measles epidemic was declared eradicated, thanks to these robust molecular epidemiological data. However, sporadic measles cases and small outbreaks have consistently been found in Japan, even in the post-elimination era. This study examined the molecular epidemiology of MV between 2008 and 2017 on a national scale. Genotyping data indicating the termination of endemic transmission of the MV is one of the essential conditions that must be proved in order to eradicate the disease. Most measles cases in prefectural public health facilities in the country have received MV genotype analyses since 2014. These reliable molecular epidemiological data allowed Japan to declare the measles epidemic to be completely eradicated in March 2015. But even in the post-elimination era, Japan has continually had isolated cases and small outbreaks of measles. This study investigated the nationwide biological epidemiology of MV from 2008 to 2017 [22].

Measles transmission has a long history in medical facilities. Healthcare professionals who have not had the measles vaccination or who lack confirmation of immunity endanger both themselves and their patients. The research on measles vaccination laws and how they apply to healthcare providers, the disease frequency among medical care employees, the spread and the burden of measles in healthcare environments, and the impacts and expenditures related to healthcare-associated measles outbreaks in healthcare facilities were all carefully reviewed. If medical professionals cannot prove that they have received two doses of the measles vaccine or have other verification of immunity, addressing measles transmissions in healthcare settings may be challenging. It can be disruptive and expensive to assess and contain exposures and outbreaks in hospital settings. A key method for attaining measles eradication is establishing vaccination programs for healthcare workers, which should be a top priority for international policy-setting organizations, governments, and hospitals. Assessing and containing exposure and outbreaks in a hospital context can be unpleasant and expensive. Establishing immunization programs for healthcare professionals is a crucial strategy for eradicating measles, and it should be the most important thing for international policy-setting bodies, governments, and hospitals [23].

Outbreak in California

The 2014-2015 Disneyland measles epidemic, which started at the Californian amusement park in December 2014, ignited debates about measles, vaccine reluctance, and vaccination policy throughout the globe. The outbreak coincided with rising patterns in non-medical vaccination exceptions in California and elsewhere, and it drew to a close a year that saw the largest number of measles cases documented in 20 years. The epidemic attracted a lot of media attention because of its dramatic plot and spread among unprotected communities, with the emphasis on vaccination hesitancy as the leading cause of the outbreak [24].

Even though the elimination of measles is scientifically and physiologically possible, it is being held back by poor immunization program results, a lack of political will, overly cautious international organizations, and a lack of donor priority [25]. In the years following its eradication, a sizable majority of cases in the US included people who had purposefully avoided vaccination. People who reject vaccinations and those who have received all recommended vaccinations are both at an elevated risk of contracting measles, according to the phenomena of vaccine refusal. Although declining immunity and other reasons have been implicated in the pertussis revival, vaccination rejection was still linked to an elevated risk of developing pertussis in some communities [26].

Outbreak in the USA

Due to an amalgam of increasing international travel and lower vaccination rates across the nation, measles, which was formerly an uncommon illness in the US, is reappearing in our communities. As healthcare professionals, we must familiarize ourselves with this illness so that we may accurately identify it and instruct those we treat on how to avoid it [27].

A measles outbreak that affected the whole US army in 1917-1918 resulted in over 95,000 illnesses along with more than 3000 fatalities. In addition to identifying a concurrent pandemic of the primary streptococcal pneumonia in troops without measles, an outbreak investigation linked rubella and streptococcal co-infections to the majority of deaths. Findings from the outbreak are still crucial for comprehending and treating severe pneumonia medically [28]. Medical facilities were required by the stringent enactment of the Occupational Health and Safety Act to determine medical staff resistance to measles and to vaccinate those with susceptible pockets during the private sector measles outbreak in Korea in 2019. Despite receiving two doses of the measles, mumps, and rubella vaccine, measles occurred in healthcare employees during this outbreak [29].

Since 2000, the majority of cases in the US have been linked to visitors who contract the disease abroad and then pass it on to vulnerable populations in the country. Measles complications can cause severe morbidity and death and are rather prevalent [30]. Due to the WHO's, also called the UN International Children's Fund (UNICEF), implementation of the measles decrease in mortality plan, significant progress has been made in lowering measles incidence and death [31].

Vaccination drive for the eradication of measles

The years have shown that vaccination campaigns are efficient and helpful in preventing infectious diseases. However, given current trends, which indicate that many developing nations will not meet the Millennium Development Goals (MDG), there is an urgent need to step up efforts to control the most prevalent illnesses that are still the leading causes of mortality and morbidity in children under the age of five, such as diarrhea and pneumonia, since effective and secure vaccines are now available [32].

The endemic transmission of the MV was finally eradicated in the US in September 2016, making it the first region in the world to achieve this. In addition, a number of other nations have confirmed the eradication of measles, and countries in all six regions of the WHO have set measles elimination targets. These elimination targets involve achieving high vaccination coverage, strengthening immunization systems, enhancing surveillance, and responding rapidly to outbreaks. These efforts aim to eventually eliminate measles as a public health threat globally. Therefore, more nations are beginning to utilize the public health measures used to address measles epidemics in elimination contexts [33].

As a result of some youngsters receiving their vaccinations either late or never, efforts to eliminate measles in the European Union (EU) and the European Economic Area (EEA) by 2015 are stymied. Migrants are one group that is known to have a higher risk of contracting measles; however, it is unclear to what degree this is the situation due to a shortage of statistics [34]. Globally, significant strides have been achieved to lessen the burden of measles-related infant mortality, and measles cases have sharply declined as two doses of the measles-containing vaccination have been administered to more people [35-36].

## Conclusions

The results of a measles outbreak in socioeconomically diverse sections can vary significantly. In areas with high socioeconomic status, generally, higher vaccination coverage leads to fewer cases and more rapid containment of outbreaks. These areas might experience less severe impacts on public health systems and lower overall costs related to managing the outbreak. In areas with low socioeconomic status, lower vaccination rates often result in higher transmission rates and more severe outbreaks. These areas may face greater public health challenges, including higher morbidity and mortality rates, increased healthcare costs, and significant disruptions to daily life and local economies. Additionally, limited access to healthcare and educational resources can exacerbate the outbreak's effects.

Overall, the socioeconomic diversity within a region can lead to varying impacts and outcomes during a measles outbreak, highlighting the need for targeted public health interventions to address these disparities. The measles outbreak in areas with lower socioeconomic standing highlights the urgent need for all-encompassing initiatives that focus on the social and economic determinants of health, enhance access to healthcare, and support immunization. We can strive for a fairer and healthier society where the impact of infectious diseases is minimized for all individuals, irrespective of their socioeconomic status, by prioritizing these actions.

## References

[1] Moss WJ (2017). Measles. Lancet.

[2] Lievano F, Galea SA, Thornton M (2012). Measles, mumps, and rubella virus vaccine (M-M-R™II): a review of 32 years of clinical and postmarketing experience. Vaccine.

[3] Jit M, Dang TT, Friberg I (2015). Thirty years of vaccination in Vietnam: impact and cost-effectiveness of the national Expanded Programme on Immunization. Vaccine.

[4] Hübschen JM, Gouandjika-Vasilache I, Dina J (2022). Measles. Lancet.

[5] Gupta SN, Gupta N (2009). Two highly immunized hilly areas versus double measles outbreak investigations in District Kangra, Himachal Pradesh, India, in 2006. J Glob Infect Dis.

[6] Singh J, Kumar A, Rai RN (1999). Widespread outbreaks of measles in rural Uttar Pradesh, India, 1996: high risk areas and groups. Indian Pediatr.

[7] Bharti B, Bharti S (2002). Measles in a hilly hamlet of northern India. Indian J Pediatr.

[8] Moss WJ, Griffin DE (2012). Measles. Lancet.

[9] Phadke VK, Bednarczyk RA, Salmon DA, Omer SB (2016). Association between vaccine refusal and vaccine-preventable diseases in the United States: a review of measles and pertussis. JAMA.

[10] Griffin DE (2018). Measles vaccine. Viral Immunol.

[11] Dubé E, Gagnon D, MacDonald NE (2015). Strategies intended to address vaccine hesitancy: review of published reviews. Vaccine.

[12] Rota PA, Moss WJ, Takeda M, de Swart RL, Thompson KM, Goodson JL (2016). Measles. Nat Rev Dis Primers.

[13] Nandy R, Handzel T, Zaneidou M (2006). Case-fatality rate during a measles outbreak in eastern Niger in 2003. Clin Infect Dis.

[14] Leung AK, Hon KL, Leong KF, Sergi CM (2018). Measles: a disease often forgotten but not gone. Hong Kong Med J.

[15] Bose AS, Rai P, Gupta BP (2022). Nepal measles outbreak response immunization during COVID-19: a risk-based intervention strategy. Vaccine.

[16] Basa S, Das RR, Khan JA (2015). Root-cause analytical survey for measles outbreak: vaccination or vaccine?- a study from Madhepura district, Bihar, India. J Clin Diagn Res.

[17] Dzeyie KA, Lowang D, Dikid T, Wangsu W, Tamir T (2021). Measles outbreak investigation at Indo-Myanmar border, Longding District, Arunachal Pradesh, India, 2017. Indian J Public Health.

[18] Manakongtreecheep K, Davis R (2017). A review of measles control in Kenya, with focus on recent innovations. Pan Afr Med J.

[19] Freund R, Krivine A, Prévost V (2013). Measles immunity and measles vaccine acceptance among healthcare workers in Paris, France. J Hosp Infect.

[20] Angelo KM, Gastañaduy PA, Walker AT, Patel M, Reef S, Lee CV, Nemhauser J (2019). Spread of measles in Europe and implications for US travelers. Pediatrics.

[21] Santibanez S, Hübschen JM, Muller CP (2015). Long-term transmission of measles virus in Central and continental Western Europe. Virus Genes.

[22] Seki F, Miyoshi M, Ikeda T (2019). Nationwide molecular epidemiology of measles virus in Japan between 2008 and 2017. Front Microbiol.

[23] Doll MK, Correira JW (2021). Revisiting the 2014-15 Disneyland measles outbreak and its influence on pediatric vaccinations. Hum Vaccin Immunother.

[24] Doll MK, Weitzen SD, Morrison KT (2021). Trends in the uptake of pediatric measles-containing vaccine in the United States: a Disneyland effect?. Vaccine.

[25] Feikin DR, Lezotte DC, Hamman RF, Salmon DA, Chen RT, Hoffman RE (2000). Individual and community risks of measles and pertussis associated with personal exemptions to immunization. JAMA.

[26] Durrheim DN, Crowcroft NS, Strebel PM (2014). Measles - the epidemiology of elimination. Vaccine.

[27] Durrheim DN (2020). Measles eradication-retreating is not an option. Lancet Infect Dis.

[28] Morens DM, Taubenberger JK (2015). A forgotten epidemic that changed medicine: measles in the US Army, 1917-18. Lancet Infect Dis.

[29] Khan L (2018). Measles in children. Pediatr Ann.

[30] Schuchat A (2015). Measles in our time: the US experience. Future Virol.

[31] Seok H, Park DW, Kim KN (2021). Report of the Korean Society of Infectious Diseases Roundtable Discussion on responses to the measles outbreaks in Korea in 2019. Infect Chemother.

[32] Alves Graber EM, Andrade FJ Jr, Bost W, Gibbs MA (2020). An update and review of measles for emergency physicians. J Emerg Med.

[33] Moss WJ (2009). Measles control and the prospect of eradication. Curr Top Microbiol Immunol.

[34] Williams GA, Bacci S, Shadwick R (2016). Measles among migrants in the European Union and the European Economic Area. Scand J Public Health.

[35] Chauke-Moagi BE, Mumba M (2012). New vaccine introduction in the East and Southern African sub-region of the WHO African region in the context of GIVS and MDGs. Vaccine.

[36] Gastañaduy PA, Banerjee E, DeBolt C (2018). Public health responses during measles outbreaks in elimination settings: strategies and challenges. Hum Vaccin Immunother.

